# Cardiometabolic factors and vitamin D deficiency in pediatric patients with chronic kidney disease

**DOI:** 10.3389/fnut.2024.1480424

**Published:** 2024-10-08

**Authors:** Israel Parra-Ortega, Jessie Nallely Zurita-Cruz, Miguel Angel Villasis-Keever, Miguel Klünder-Klünder, Jenny Vilchis-Gil, Carmen Zepeda-Martinez, Ángeles Rizo Romero, Gabriela Alegria-Torres, Benjamin Romero-Navarro, José Carlos Romo-Vázquez

**Affiliations:** ^1^Auxiliary Diagnostic Services, Hospital Infantil de México Federico Gómez, Ministry of Health (SSA), Mexico City, Mexico; ^2^Facultad de Medicina Universidad Nacional Autónoma de México, Hospital Infantil de México Federico Gómez, Ministry of Health (SSA), Mexico City, Mexico; ^3^Analysis and Synthesis of the Evidence Research Unit, National Medical Center XXI Century, Instituto Mexicano del Seguro Social, Mexico City, Mexico; ^4^Epidemiological Research Unit in Endocrinology and Nutrition, Hospital Infantil de México Federico Gómez, Ministry of Health (SSA), Mexico City, Mexico; ^5^Department of Pediatric Nephology, Children’s Hospital, National Medical Center XXI Century, Instituto Mexicano del Seguro Social, Mexico City, Mexico; ^6^Department of Pediatric Nephology, Hospital Infantil de México Federico Gómez, Ministry of Health (SSA), Mexico City, Mexico

**Keywords:** dyslipidemia, vitamin D, chronic kidney disease, pediatric, cardiometabolic factors

## Abstract

**Background:**

Patients with chronic kidney disease (CKD) are at increased risk for cardiovascular disease. Up to 80% of patients with CKD may exhibit inadequate vitamin D (VD) levels, which have been linked to the presence of cardiometabolic factors (CFs) in the adult population. However, research on this association in the pediatric population is limited.

**Objective:**

To analyze the effects of 25-hydroxyvitamin D3 (25-[OH]D) levels and status on the presence of CFs in children receiving kidney replacement therapy (KRT).

**Materials and methods:**

This cross-sectional study included pediatric patients receiving KRT, aged 8–17 years, who were receiving hemodialysis or peritoneal dialysis from January 2021 to March 2024. We conducted anthropometric measurements, blood pressure assessments, and glucose, 25-(OH)D, and lipid profiling for all participants. The daily dose of cholecalciferol supplementation, as well as other medications affecting bone and lipid metabolism and antihypertensive drugs, were documented. Statistical analyses were performed using Student’s *t*-tests and chi-square tests to compare the CFs between groups with and without VD deficiency.

**Results:**

The study involved 156 patients with an average age of 12.9 years and a mean serum VD level of 22.5 ng/dL. Patients with VD deficiency presented higher levels of total cholesterol and diastolic blood pressure (*p* < 0.05). No statistically significant differences were found in other biochemical profile variables or in the frequency of cardiometabolic factors.

**Conclusion:**

Vitamin D deficiency seems to increase the risk of dyslipidemia and uncontrolled hypertension in children and adolescents with end-stage CKD.

## 1 Introduction

Vitamin D (VD) is a lipid-soluble steroid hormone equipped with a distinct cytosolic receptor. While initially associated with the metabolism of calcium and phosphorus, recent discoveries highlight its broader role in affecting multiple key extraskeletal systems across various target organs, including fat tissues, blood cells, immune components, skin, muscles, the pancreatic endocrine system, and vascular structures (1, 2). The VD receptor (VDR) is found in virtually all body organs and functions through both genomic (nuclear VDR) and nongenomic (membrane VDR) mechanisms. The majority of human VD is derived from the synthesis in the skin induced by sunlight (approximately 80%), with the remainder obtained from dietary intake and supplements (3, 4). The factors contributing to VD deficiency include dark skin, inactive lifestyles, inadequate sun exposure, environmental pollution, obesity, and insufficient VD supplementation (5).

In the context of chronic kidney disease (CKD)-related mineral and bone disorders, VD is crucial because of the presence of 1-*α* hydroxylase in the kidneys, which is pivotal for bone formation and resorption. Deficient levels of 25-hydroxyvitamin D3 (25-[OH]D) in the serum can lead to a negative calcium balance, induce secondary hyperparathyroidism, and result in bone disorders. In CKD, the increase in hyperphosphaturic osteocyte-derived hormone (FGM-23) acts to counter phosphate retention, which in turn suppresses renal 1α-hydroxylase expression and promotes 24-hydroxylase expression and results in the degradation of 1,25(OH)D. Impaired 25(OH)D absorption due to kidney disease is the predominant cause of 1,25(OH)D deficiency (3). Consequently, patients with end-stage CKD often lack both activated and nutritional VD (6).

Considering that a serum 25(OH)D level < 20 ng/mL signifies a deficiency and that >30 ng/mL is necessary for optimal health, both the Kidney Disease Outcomes Quality Initiative (KDOQI) and Kidney Disease Improving Global Outcomes (KDIGO) suggest annual testing of 25(OH)D levels for children with CKD stages 2–5 and advocate for supplementation when levels fall below 30 ng/mL (7). Additionally, the KDOQI guidelines recommend the use of cholecalciferol to address VD insufficiency in CKD stages 3 and 4 and active VD hormone treatments for VD deficiency in patients with stage 5 CKD who also exhibit secondary hyperparathyroidism, with observations showing that 25(OH)D inadequacy continues as CKD progresses from stages 3 to 5 (8).

Cardiovascular deaths in adults with CKD are due primarily to coronary artery disease and congestive heart failure. In contrast, the leading causes of death within the pediatric population include arrhythmias, valvular diseases, cardiomyopathy, and cardiac arrest. This distinction highlights the varying impact of cardiovascular complications across different age groups with CKD (9, 10).

Endothelial dysfunction, an early indicator of cardiac issues, manifests in the initial stages of CKD in both children and adults (9). Recent studies focusing on the pediatric population have revealed a high incidence and prevalence of cardiovascular risk factors associated with CKD. These findings underscore the importance of early detection and management of endothelial health in young patients to mitigate long-term cardiovascular complications (11).

Previous studies in adults have demonstrated significant associations between vitamin D deficiency and an increased risk of cardiometabolic factors (CFs), including metabolic syndrome. For example, among adult patients on hemodialysis, the prevalence of metabolic syndrome increased as vitamin D levels decreased, with the highest prevalence observed in those with 25(OH)D levels below 20 ng/mL. These patients also exhibited negative associations between vitamin D and factors such as diastolic blood pressure and triglyceride levels (12). Further studies indicate that low levels of 25(OH)D are associated with components of metabolic syndrome, such as central obesity, hypertension, and dyslipidemia (13). In contrast, no significant associations between 25(OH)D and metabolic syndrome were observed in patients on peritoneal dialysis, suggesting possible differences related to the type of CKD treatment (14). Additionally, the implications of elevated parathyroid hormone (PTH) and its relationship with lipogenesis and obesity are discussed (15).

Clinical trials and meta-analyses have shown a potentially beneficial effect of vitamin D supplementation on reducing total serum cholesterol, LDL cholesterol, and triglyceride levels in patients with hypercholesterolemia and vitamin D insufficiency (16, 17).

Research in the pediatric population suggests an association between vitamin D deficiency and risk factors for metabolic syndrome, including increased HDL cholesterol and the mitigation of triglyceride increases (18). Specifically, 2,596 students with a mean age of 12.2 years were studied, and it was demonstrated that those with deficient vitamin D levels had higher odds of Metabolic Syndrome (OR: 4.25), abdominal obesity (OR: 2.24), low HDL-C (OR: 1.65), and high fasting blood sugar (OR: 2.56) compared to those with sufficient vitamin D levels (19). Recent studies have shown an association between vitamin D deficiency and cardiovascular diseases during the progression of CKD (20). A study of 34 children with CKD revealed that vitamin D levels are inversely correlated with increases in the left ventricular mass index (*r* = −0.5; *p* < 0.05) (21).

These findings suggest that the relationship between vitamin D and CFs may vary depending on the population and the status of kidney disease, highlighting the need to explore these links in children with CKD to better understand their impact on this young population.

Therefore, our study aimed to analyze the effects of the level and status of 25-hydroxyvitamin D3 (25-[OH]D) on the presence of CFs in children with CKD.

## 2 Materials and methods

### 2.1 Subjects

This cross-sectional study was conducted from January 2021 to March 2024 at two tertiary pediatric care centers in Mexico City. We enrolled children aged 6 to 18 years who were diagnosed with stage 5 CKD, as classified by the National Kidney Foundation’s Kidney Disease Outcomes Quality Initiative (NKF-K/DOQI) (22). These children were receiving replacement therapy with either peritoneal dialysis or hemodialysis. Patients with a family history of dyslipidemia, hepatic insufficiency, or those receiving steroid therapy were excluded from the study.

Among the 169 potential candidates for inclusion, 13 were excluded (5 children were younger than 6 years, 3 underwent kidney transplants less than 3 months previously, and 11 declined to participate in the study). The final number of participants included was 156 patients.

In accordance with the Declaration of Helsinki, the protocol was evaluated and approved by the ethics and research committee of the hospital with registry numbers R-2010-3603-7, R-2023-785-096, and HIM-2017-117. The parent or legal guardian signed a written informed consent form, and each child provided written assent according to the recommendations of the Declaration of Helsinki.

### 2.2 Variables

The patient records provided demographic data, the type of renal replacement therapy (dialysis or hemodialysis), the etiology of chronic kidney disease (CKD), duration since diagnosis, diagnosis of hypertension, cholecalciferol supplementation and dosage, and use of calcitriol, lipid-lowering agents, phosphate binders, calcimimetics, and antihypertensives. To determine and quantify the adequacy of hemodialysis and peritoneal dialysis treatments, the Kt/V was calculated (K, dialyzer clearance of urea; t, dialysis time; V, volume of distribution of urea). Hemodialysis was deemed adequate when Kt/V exceeded 1.2 per week, and peritoneal dialysis was considered adequate when Kt/V was greater than 1.8 per week (22, 23).

#### 2.2.1 Anthropometry and blood pressure

The anthropometric indicators of each patient were recorded by a certified nutritionist. Height was measured to the nearest 0.1 cm with a SECA Model 769 stadiometer (SECA 769, SECA Corp. Oakland Center Columbia, MD, United States). Weight measurements were conducted using the bioimpedance method (Tanita BC-568 Segmental Body Composition Monitor, Tokyo, Japan), with patients barefoot and wearing underwear. Body mass index (BMI) was calculated by dividing the weight in kilograms by the height in meters squared, and then the percentile and BMI z score were obtained according to age and sex. Classification of BMI was defined by the Centers for Disease Control and Prevention 2000, with children considered obese when their BMI for age and sex was in the ≥95th percentile, overweight when their BMI was >85th but <95th percentile, malnourished when their BMI was < 25th percentile, and normal weight when their BMI was within the 25th and 84th percentiles (24). Blood pressure was measured with auscultatory methods using a mercury sphygmomanometer according to age in duplicate and reported as a percentile according to age, sex, and height. Measurements were taken with the participant sitting in a chair with their feet flat on the floor and their back supported after a 10-min rest period in the hospital (25, 26).

#### 2.2.2 Blood analysis

Blood samples were obtained from the forearm of each subject via the antecubital vein between 7:00 and 8:00 a.m., and after a minimum of 12 h of fasting. Serum samples were frozen at −80°C until analysis. Glucose, triglycerides (TGLs), high-density lipoprotein cholesterol (HDLc), urea, creatinine, and parathyroid hormone levels were determined by colorimetric enzymatic methods (Bayer Diagnostics, Puteaux, France). Intra- and interassay coefficients of variation <7% were considered acceptable. A standard curve was also generated for each assay. For LDL-C, we utilized DeLong’s modified Friedwald formula (24).

#### 2.2.3 Cardiometabolic profile (definition)

Children with diastolic or systolic blood pressure ≥ the 90th percentile for age and sex, according to the National Blood Pressure Education Program Working Group were considered to have hypertension (26). Obesity was indicated by a BMI ≥ the 95th percentile, and overweight was indicated by a BMI ≥ the 85th percentile for age and sex according to the 2000 CDC Growth Charts (27). For patients whose height was more than 2 standard deviations less than average for age, the BMI z score was adjusted for height and age. A fasting glucose level ≥ 100 mg/dL was considered elevated (27). Hypertriglyceridemia was assessed TGLs ≥90th percentile for age and sex for children <10 years old and TGLs ≥150 mg/dL for children >10 years old (27, 28). LDL hypercholesterolemia was assessed as LDLc ≥90th percentile for age and sex for children <10 years old and LDLc ≥130 mg/dL for children >10 years old. Reduced HDLc was defined as HDLc <10th percentile for age and sex for children <10 years old and HDLc <40 mg/dL in males and < 50 mg/dL in females for children >10 years old, according to the International Diabetes Federation (IDF) definition (27, 28). Dyslipidemia was defined as the presence of hypertriglyceridemia, reduced HDLc or LDL hypercholesterolemia. Hypertension was assessed using systolic and diastolic blood pressure according to age. In patients <13 years, systolic or diastolic blood pressure ≥ 95th percentile for age, height, and sex was considered hypertensive. For those >13 years old, those whose systolic blood pressure was ≥130 mmHg or whose diastolic blood pressure was ≥80 mmHg (29) were considered hypertensive if their medical records indicated a diagnosis and if they were taking antihypertensive medication to manage it. Cases of uncontrolled hypertension were identified when, despite the use of antihypertensives, physical examinations revealed elevated blood pressure levels based on the previously mentioned criteria.

### 2.3 Vitamin D determination

The serum concentrations of 25(OH)D (25-hydroxyvitamin D3) were measured using the Abbot chemoluminescence technique with Archirech 1,000 equipment. A serum level of <20 ng/mL was considered VD deficiency, 20–29.99 ng/mL was considered insufficient, and > 30 ng/mL was considered normal (30).

### 2.4 Statistical analyses

For quantitative variables, Kolmogorov–Smirnov tests were performed to evaluate the normality of the distribution. The quantitative variables were not normally distributed, so they are presented as the means and standard errors. The qualitative variables are presented as proportions and frequencies. While the Endocrine Society classifies vitamin D status into three categories (deficiency <20 ng/mL, insufficiency 20–29.99 ng/mL, and normal >30 ng/mL) (31). Initially, the patients were compared across these three groups, but no statistically significant differences were found in the multivariate models. Studies that have found links between vitamin D levels and cardiometabolic factors often use a two-group classification: deficiency (<20 ng/mL) and no deficiency (>20 ng/mL) (17, 32). Accordingly, this study grouped patients into two categories: those with vitamin D deficiency and those without. A sub-analysis was conducted according to age groups: school-aged children (6–10 years, *n* = 36) and adolescents (11–18 years, *n* = 120). Logarithmic transformation of the quantitative variables was performed for statistical tests. The comparisons of qualitative variables between the groups were performed using the chi-square test and the Student’s *t*-test was used for quantitative variables. To evaluate the correlation of biochemical parameters with serum VD levels, the Spearman test was used. A multiple linear regression model was used to evaluate the association of serum vitamin D levels with LDL cholesterol and adjusted for confounding variables including age, sex, body mass index z score, cholecalciferol supplementation dose, and replacement treatment. A multiple logistic regression model was used to evaluate the association of vitamin D deficiency with the presence of dyslipidemia and uncontrolled hypertension with adjustments for confounding variables (age, parathyroid hormone, obesity, and cholecalciferol supplementation dose). The model was constructed considering subjects who had one or more cardiometabolic factors (dyslipidemia and hypertension) in order to make the model more robust. A value of *p* < 0.05 was considered statistically significant. STATA software (Stata Corp, College Station, TX, United States), version 12.0, was used for the statistical analyses.

## 3 Results

Of the 156 participants, the average age was 12.9 years, 51.9% were female, and 78.8% were classified as normal weight based on their BMI z score. The most common etiology of CKD was congenital anomalies of the kidney and urinary tract (CAKUT), which were observed in 47.4% of cases. The modalities of renal replacement therapy, peritoneal dialysis, and hemodialysis were distributed in similar proportions, with only 10.9% showing inadequate dialysis (Table 1). The average level of 25-hydroxyvitamin D was 22.5 ng/dL. Vitamin D deficiency was present in 21.2% (*n* = 33) of the patients, whereas nearly half (48.7%, *n* = 76) had sufficient levels of VD (Table 1).

**Table 1:** Characteristics of pediatric patients with chronic kidney disease included in the study.

Characteristics	Total *n* = 156
Age, y	
Mean (Standard Error)	12.9 (0.25)
Sex, %	
Female	81 (51.9)
Male	75 (48.1)
Anthropometry, mean (standard error)	
Weight, kg	35.6 (1.07)
Height, cm	139.2 (1.61)
Height-for-age z score	−2.6 (0.14)
Body mass index, kg/m^2^	17.6 (0.27)
Body mass index z score	−0.93 (0.11)
Nutritional status, n (%)	
Normal (25th and 84th pc)	123 (78.8)
Malnutrition (< 25th pc)	27 (10.9)
Overweight/obesity (≥85th pc)	16 (10.3)
CKD etiology, n (%)	
CAKUT 4	74 (47.4)
Glomerulopathy	33 (21.2)
Tubulopathy	2 (1.3)
Immunological	6 (3.8)
Indeterminate	41 (26.3)
Replacement treatment; n (%)	
Hemodialysis	75 (48.1)
Peritoneal dialysis	81 (51.9)
Time of renal replacement, y	
Mean (Standard Error)	3.8 (0.28)
Kt/V altered, n (%)	
Yes	17 (10.9)
25(OH)D, ng/mL	
Mean (Standard Error)	22.5 (0.97)
Vitamin D status, %	
<20 ng/mL (deficiency)	76 (48.7)
20–29.9 ng/mL (insufficiency)	47 (30.1)
≥30 ng/mL (normal)	33 (21.2)

25(OH)D, 25-hydroxyvitamin D3; CAKUT, congenital anomalies of the kidney and the urinary tract; CKD, chronic kidney disease.

Table 2 presents a comparison of lipid profiles and cardiometabolic factors according to whether children had vitamin D deficiency (Table 2). In terms of the biochemical profile, the average glucose level was 90 mg/dL, and the total cholesterol level was 162.9 mg/dL. The most frequent cardiometabolic alterations related to the biochemical profile were hypertriglyceridemia (42.9%, *n* = 67) and low HDL cholesterol levels (42.3%, *n* = 66), with 66.7% of the patients presenting with dyslipidemia. Additionally, 57.0% (*n* = 89) of the patients had hypertension, 53 of whom had uncontrolled hypertension.

**Table 2:** Comparison of lipid profiles and cardiometabolic factors according to vitamin D deficiency and sufficiency in pediatric patients with chronic kidney disease (CKD) included in the study.

Characteristics	All *n* = 156	Vitamin D deficiency		*p* ^*^
		With *n* = 76	Without *n* = 80	
Biochemical profile and blood pressure, mean (standard error)				
Glucose, mg/dL	90.0 (1.58)	91 0.6 (2.40)	88.5 (2.07)	0.162
Total cholesterol, mg/dL	162.9 (3.79)	174.5 (6.48)	151.9 (3.73)	*0.001*
HDL cholesterol, mg/dL	47.7 (1.11)	47.6 (1.55)	47.8 (1.60)	0.467
LDL cholesterol, mg/dL	82.9 (3.01)	90.6 (4.89)	75.6 (3.41)	*0.006*
Triglycerides, mg/dL	155.8 (6.46)	163.9 (9.66)	148.2 (8.61)	0.112
Systolic blood pressure, mmHg	112.6(1.33)	112.8 (1.79)	112.3 (1.97)	0.423
Diastolic blood pressure, mmHg	72.4 (1.08)	74.4 (1.50)	70.4 (1.53)	*0.033*
Parathormone, pg./mL	629.1 (50.3)	638.1 (70.0)	620.6 (72.5)	0.431
Cardiometabolic factors, n (%)				
Hyperglycemia	33 (21.1)	18 (23.7)	15 (18.7)	0.451
Decreased HDLc	66 (42.3)	37 (48.7)	29 (36.2)	0.116
LDL hypercholesterolemia	13 (8.3)	8 (10.5)	5 (6.2)	0.334
Hypertriglyceridemia	67 (42.9)	35 (46.1)	32 (40.0)	0.445
Dyslipidemia	104 (66.7)	56 (73.7)	48 (60.0)	0.070
Hypertension	89 (57.0)	48 (63.2)	41 (51.2)	0.133
Uncontrolled hypertension	53 (34.0)	31 (40.8)	22 (27.5)	0.080
Dyslipidemia or uncontrolled hypertension	118 (75.6)	63 (82.9)	55 (68.7)	*0.040*
Confounding factors, n (%)				
Hyperparathyroidism	92 (59.0)	49 (64.5)	43 (53.7)	0.173
Use of phosphate binders	112 (73.2)	58 (76.3)	56 (70.0)	0.374
Use of cholecalciferol supplementation	79 (50.6)	24 (31.6)	55 (68.7)	*0.001*
Cholecalciferol supplementation dose, UI, mean (standard error)	982.0 (112.2)	168.4 (41.0)	1755.0 (176.4)	*0.001*
Use of calcitriol	82 (73.9)	48 (77.4)	34 (69.4)	0.339
Use of lipid-lowering agents	25 (16.0)	15 (19.7)	10 (12.5)	0.218
Obesity	11 (7.0)	4 (5.3)	7 (8.7)	0.395

*Student’s *t-*test for independent data and chi^2^ Pearson.The values in italics indicate a significance level of *p* < 0.05.

A comparison of the biochemical profiles of patients with VD deficiency (*n* = 76) and without (*n* = 80) VD deficiency revealed that those with deficiency presented higher total cholesterol levels (174.5 mg/dL vs. 151.9 mg/dL, *p* = 0.001), LDL cholesterol levels (163.9 mg/dL vs. 148.2 mg/dL, *p* = 0.006), and diastolic blood pressure (74.4 mmHg vs. 70.4 mmHg, *p* = 0.033). When comparing the lipid profile and cardiometabolic alterations between patients with and without vitamin D deficiency across different age groups, it was observed that both school-aged children and adolescents with vitamin D deficiency had higher total cholesterol levels compared to those without the deficiency. As for hypertriglyceridemia, a higher proportion was found only in vitamin D-deficient school-aged children (80.0% vs. 42.9%) (Table 3).

**Table 3:** Comparison of lipid profiles and cardiometabolic factors between patients with vitamin D deficiency and sufficiency according to age group in pediatric patients with chronic kidney disease (CKD) included in the study.

Characteristics	Vitamin D deficiency		p*	Vitamin D deficiency		p*
	With *n* = 15	Without *n* = 21		With *n* = 61	Without *n* = 59	
Biochemical profile and blood pressure, mean (standard error)	School-aged children (6–10 y) (*n* = 36)			Adolescents (11–18y) (*n* = 120)		
Glucose, mg/dL	100.7 (7.62)	89.2 (4.19)	0.077	89.2 (2.28)	88.2 (2.40)	0.579
Total cholesterol, mg/dL	213.3 (6.48)	165.1 (8.46)	*0.015*	*165.0 (6.33)*	*147.1 (3.93)*	*0.041*
HDL cholesterol, mg/dL	50.5 (3.19)	50.1 (3.91)	0.961	46.9 (1.68)	47.0 (1.85)	0.674
LDL cholesterol, mg/dL	118.2 (16.08)	83.9 (7.42)	0.112	83.8 (4.31)	72.6 (3.76)	0.068
Triglycerides, mg/dL	214.3 (20.29)	158.6 (17.26)	0.044	151.5 (10.4)	144.5 (9.98)	0.569
Systolic blood pressure, mmHg	103.2 (3.63)	104.5 (4.18)	0.834	115.2 (|0.94)	115.1 (2.14)	0.769
Diastolic blood pressure, mmHg	68.3 (2.46)	64.6 (3.01)	0.628	75.9 (1.72)	72.5 (1.71)	0.200
Parathormone, pg./mL	517.0 (168.0)	650.4 (151.6)	0.596	667.8 (77.1)	610.0 (83.0)	0.280
Cardiometabolic factors, n (%)						
Hyperglycemia	5 (33.3)	7 (33.3)	1.000	13 (21.3)	8 (13.6)	0.264
Decreased HDLc	5 (33.3)	7 (33.3)	1.000	31 (52.5)	22 (37.3)	0.095
LDL hypercholesterolemia	4 (26.7)	2 (9.5)	0.174	4 (6.6)	3 (5.1)	0.731
Hypertriglyceridemia	*12 (80.0)*	*9 (42.9)*	*0.026*	23 (37.7)	23 (38.9)	0.886
Dyslipidemia	13 (86.7)	13 (61.9)	0.102	43 (70.5)	35 (59.3)	0.200
Hypertension	8 (53.3)	11 (52.4)	0.955	40 (65.6)	30 (50.8)	0.102
Uncontrolled hypertension	6 (40.0)	5 (23.8)	0.298	25 (41.0)	17 (28.8)	0.162
Dyslipidemia or uncontrolled hypertension	15 (71.4)	13 (86.7)	0.278	50 (82.0)	40 (67.8)	0.073
Confounding factors, n (%)						
Hyperparathyroidism	7 (46.7)	12 (57.1)	0.535	42 (68.8)	31 (52.5)	0.067
Use of phosphate binders	12 (80.0)	17 (80.9)	0.943	46 (75.4)	39 (66.1)	0.262
Use of cholecalciferol supplementation	8 (53.3)	14 (66.7)	0.418	*16 (26.2)*	*41 (69.5)*	*<0.001*
Cholecalciferol supplementation dose, UI, mean (standard error)	*168.4 (41.0)*	*1755.0 (176.4)*	*0.035*	*157.3 (48.8)*	*1796.6 (205.6)*	*<0.001*
Use of calcitriol	12 (80.0)	13 (61.9)	0.245	43 (70.5)	33 (55.9)	0.098
Use of lipid-lowering agents	3 (20.0)	4 (19.0)	0.943	12 (19.7)	6 (10.2)	0.145
Obesity	2 (9.5)	1 (6.7)	0.760	3 (4.9)	5 (8.5)	0.435

The values in italics indicate a significance level of *p* < 0.05.

The serum concentrations of each biochemical parameter were correlated with the serum vitamin D concentration, revealing a weak negative correlation with total cholesterol and LDL cholesterol (*p* < 0.05). When performing the sub-analysis of the correlation according to age group, the correlations with total cholesterol and LDL cholesterol remained consistent (Table 4).

**Table 4:** Correlation between levels of 25-hydroxyvitamin D3 and biochemical profile, blood pressure and parathormone in pediatric patients with chronic kidney disease (CKD).

	All (*n* = 156)		School-aged children (6–10 y) (*n* = 36)		Adolescents (11–18 y) (*n* = 120)	
Characteristics	*r*	p*	*r*	p*	*r*	p*
Glucose, mg/dL	−0.044	0.583	−0.183	0.284	0.004	0.961
Total cholesterol, mg/dL	*−0.264*	*0.001*	*−0.385*	*0.020*	*−0.260*	*0.004*
HDL cholesterol, mg/dL	−0.113	0.160	0.0001	0.999	−0.168	0.065
LDL cholesterol, mg/dL	*−0.212*	*0.007*	*−0.293*	*0.049*	*−0.214*	*0.018*
Triglycerides, mg/dL	−0.107	0.181	−0.274	0.104	−0.072	0.429
Systolic blood pressure, mmHg	0.049	0.536	−0.043	0.802	0.124	0.176
Diastolic blood pressure, mmHg	−0.068	0.393	−0.239	0.159	0.012	0.896
Parathormone, pg./mL	0.004	0.959	0.073	0.668	−0.018	0.844

*Spearman correlation.The values in italics indicate a significance level of *p* < 0.05.

The frequency of cardiometabolic factors such as dyslipidemia and uncontrolled hypertension was also significantly greater in patients with VD deficiency (82.9% versus 68.7%, *p* = 0.040). The use of cholecalciferol supplementation was greater in patients without vitamin D deficiency (68.7% vs. 31.6%, *p* = 0.001), as were the daily doses they consumed (1755.0 IU/day vs. 168.4 IU/day, *p* = 0.001). There were no differences in the effects of the other medications on bone metabolism between patients with and without VD deficiency.

Regarding the use of drugs that affect bone metabolism, 73.2% used phosphate binders (calcium carbonate, sevelamer), 50.6% used cholecalciferol with an average daily dose of 982.0 IU, and 73.9% consumed calcitriol at doses of 0.15–0.25 mcg/kg/day, with none using calcimimetics. Nearly two-thirds of the patients suffered from hyperparathyroidism (59.0%) (Table 2).

In the multivariate linear analysis, a negative association was found between VD levels (B −0.600, 95% CI −1.109 to −0.090), and a positive association was detected between peritoneal dialysis (7.751, 95% CI 1.007–14.495) and LDL cholesterol concentrations, controlling for age, sex, BMI z score, parathyroid hormone levels, and cholecalciferol supplementation (Table 5). The multiple logistic model showed that vitamin D deficiency is associated with an increased risk of dyslipidemia and uncontrolled hypertension (OR 2.2, 95% CI 1.1–4.8) when adjusted for age, obesity, parathyroid hormone levels, and cholecalciferol supplementation (Table 6).

**Table 5:** Effect of vitamin D concentrations on LDL cholesterol levels in pediatric patients with chronic kidney disease (CKD) included in the study.

Characteristics	Coefficient	CI 95%	p*
25(OH)D, ng/mL	−0.600	−1.109 to −0.090	*0.021*
Age, y	−2.613	−4.422 to −0.804	*0.005*
Male sex	6.435	−5.139 to 18.009	0.274
Body mass index, z score	1.009	−3.242 to 5.262	0.640
Cholecalciferol supplementation dose, UI	0.001	−0.004 to 0.006	0.770
Peritoneal dialysis	7.751	1.007 to 14.495	*0.005*

*Multiple linear regression model adjusted. R-squared = 0.135, *p* = 0.001.The values in italics indicate a significance level of *p* < 0.05.

**Table 6:** Association of vitamin D deficiency on the presence of dyslipidemia or uncontrolled hypertension in pediatric patients with chronic kidney disease (CKD) included in the study.

Characteristics	OR	CI 95%	p*
Vitamin D deficiency	2.212	1.007–4.856	*0.048*
Age, y	1.021	0.907–1.149	0.723
Paratohormone, pg./mL	1.000	0.999–1.001	0.057
Obesity (>95th PC)	4.865	0.560–42.236	0.151
Cholecalciferol supplementation dose, UI	0.999	0.999–1.000	0.114

*Multiple logistic regression model adjusted. R-squared = 0.063, *p* = 0.026.The values in italics indicate a significance level of *p* < 0.05.

## 4 Discussion

In the study involving patients with CKD, VD deficiency and insufficiency were observed in half of the participants. This prevalence was lower than that typically reported in this population, where rates range from 62.5% to over 80% (32, 33).

VD deficiency in patients with CKD is attributed to multiple factors, including dietary restrictions and reduced cutaneous synthesis of vitamin D due to uremia. Moreover, as renal function declines, there is a decrease in megalin, an increase in phosphate and FGF-23, and abnormalities in PTH and the retention of uremic toxins that inhibit 1α-hydroxylase activity (34, 35). This leads to reduced serum levels of calcidiol (25[OH]D) and decreased conversion to calcitriol (1,25-OH-D), causing disturbances in bone metabolism and secondary hyperparathyroidism, which was present in our patients (36).

VD is linked to cholesterol metabolism, as both share a common biosynthetic pathway. Calcitriol, the active form of VD, plays roles in remodeling and regulating inflammatory processes that drive atherosclerosis, including the proliferation of smooth muscle cells, which stabilize plaques. Calcitriol impedes cholesterol absorption by macrophages and promotes cholesterol efflux, suggesting that vitamin D metabolites may suppress foam cell formation and, consequently, atherosclerosis itself. Some interactions observed between vitamin D metabolism and cholesterol can be explained by calcidiol (25[OH]D) suppressing the activity of 3-hydroxy-3-methyl-glutaryl-coenzyme A reductase. Moreover, the vitamin D receptor induces the activity of the enzyme cholesterol 7 alpha-hydroxylase (CYP7A1), which is responsible for converting cholesterol into 7a-hydroxy cholesterol, a bile acid precursor. Specifically, regarding LDL cholesterol, VD deficiency is associated with elevated total cholesterol and LDL-C levels, and supplementation with VD could decrease total serum cholesterol and LDL-C levels (37, 38).

Building on these findings, our study observed that patients with VD deficiency presented higher levels of LDL cholesterol (163.9 mg/dL vs. 148.2 mg/dL, *p* = 0.006) than did those without VD deficiency. While similar studies in pediatric patients with CKD are lacking, research in the adult CKD population has noted that total serum cholesterol levels are higher in patients with VD deficiency than in those without deficiency (158.0 mg/dL vs. 128.6 mg/dL, *p* = 0.001). Furthermore, a two-year follow-up revealed increased mortality rates in the VD-deficient group (39% vs. 21%, *p* = 0.03) (39).

In addition to VD deficiency, various factors increase cardiometabolic risk in pediatric patients with CKD. The frequencies of dyslipidemia and hypertension can reach 83 and 56%, respectively (40, 41). In comparison, the frequency of these conditions in our study was similar, with 75% of patients with dyslipidemia and 57% with hypertension. Patients with CKD at any stage are prone to lipid abnormalities due to altered lipoprotein metabolism caused by decreased glomerular filtration. The rate of dyslipidemia is higher in patients on peritoneal dialysis, driven by increased lipoprotein synthesis in the liver due to glucose absorption from the dialysis solution, which increases insulin levels and protein loss (42, 43). Dyslipidemia may also arise due to the reduced activity of liver lipoprotein lipase and hepatic triglyceride lipase enzymes, which are influenced by uremic toxins and high levels of apoprotein C-III (22, 44). Hypertension in patients with CKD is commonly due to the inability of the kidneys to balance sodium, leading to excessive peripheral vasoconstriction (45), and hypervolemia, specifically leading to an increase in diastolic pressure (46). This condition is exacerbated by various factors, including a reduction in the number of glomeruli, sclerosis, tubular atrophy, interstitial fibrosis, inappropriate nitric oxide release, high renin–angiotensin system activity, and abnormal synthesis of polyunsaturated fatty acids and eicosanoids (45, 47). Additionally, CKD is associated with reduced insulin secretion and sensitivity (48), elevated adipokine levels such as leptin (49), and other CKD-related issues such as hyperparathyroidism and an inflammatory state, which contribute to an increased risk of hyperglycemia (50).

In this study, we found that patients with VD deficiency had higher levels of diastolic blood pressure (74.4 mmHg vs. 70.4 mmHg) and a greater proportion of uncontrolled hypertension (40.8% vs. 27.5%). While these findings have been reported in other studies, they have not been documented in pediatric patients with CKD. Several key mechanisms can explain how VD deficiency may contribute to hypertension. One mechanism involves the activation of the renin-angiotensin-aldosterone system. As plasma renin levels increase, sympathetic activity may become heightened, raising intra-glomerular pressure, which leads to elevated blood pressure, reduced glomerular filtration rate, and cardiovascular damage. VD may also have a direct impact on left ventricular hypertrophy and vascular stiffness. Low serum 25(OH)D levels can reduce nitric oxide production in blood vessels and decrease calcium influx, impairing vasodilation and contributing to increased blood pressure (51–53).

In our study, we were unable to find an association between the dose of VD supplementation and serum LDL cholesterol levels through the multivariate model, despite evidence from other studies demonstrating its effectiveness in patients with dyslipidemia (17). Based on our findings, we recommend that pediatric patients with end-stage CKD routinely have their 25(OH)D levels measured and be supplemented with cholecalciferol to maintain normal levels (>30 ng/mL). While this may not completely prevent the presence of cardiometabolic alterations, it could help reduce them to some extent. Furthermore, a clinical trial is necessary to evaluate the efficacy of vitamin D supplementation in pediatric patients with CKD and VD deficiency in lowering serum LDL cholesterol levels.

In light of these findings, it is important to acknowledge that a major limitation of this study is the temporal ambiguity resulting from the simultaneous measurement of vitamin D deficiency and cardiometabolic factors. Due to the cross-sectional design, this prevents us from establishing causality in this apparent association. Moreover, for uncontrolled hypertension, there are other factors that may contribute beyond VD deficiency, such as medication adherence, volume overload, and myocardial dysfunction, which were not measured. Another limitation is the variability in the duration of cholecalciferol supplementation among patients, which likely influenced the lack of observed benefit in the regression models.

In conclusion, vitamin D deficiency seems to increase the risk of dyslipidemia and uncontrolled hypertension in children and adolescents with end-stage CKD. These findings suggest the need to monitor vitamin D levels in order to provide appropriate supplementation when levels considered to be deficient are detected.

## Data Availability

The raw data supporting the conclusions of this article will be made available by the authors, without undue reservation.
